# Supplementary material for: Children with Chronic Immune Thrombocytopenia Exhibit High Expression of Human Endogenous Retroviruses TRIM28 and SETDB1
Source: Genes (Basel). 2023 Aug 1;14(8):1569. doi: 10.3390/genes14081569 (PMC10454145; doi:10.3390/genes14081569)
Supplement: Supplementary file 1 [file genes-14-01569-s001.zip › genes-2526377-supplementary.pdf]

Supplementary Table S1

Transcription levels of pol genes of HERV-H, HERV-K, and HERV-W; of env genes of Syncytin 1, Syncytin 2, and HERV-W; and of TRIM28 and SETDB1 in whole blood of 34 patients with chronic immune thrombocytopenia

CITP	HERV-H-pol	HERV-K-pol	HERV-W-pol	Syncitin 1	Syncitin 2	HERV-W-env	TRIM28	SETDB1	Ongoing Therapy
1	2,2148825	2,59760199	1,93638589	3,29757842	2,10602262	1,75446176	1,92729468	2,78749944	1
2	0,89373869	1,7580901	1,7323902	1,37611031	0,9659425	0,95435451	0,98847114	1,24594943	1
3	1,67813481	2,2066961	1,85154827	4,91064259	2,04878953	1,72168831	1,96854937	2,36518483	0
4	1,54694021	2,05561781	1,75044222	3,71513227	1,90050258	1,48484224	1,72573455	2,08478471	0
5	0,696952	0,917438	1,294336	1,565312	0,890125	0,604531	1,004523	0,781727	0
6	1,43764209	1,73448839	2,66638968	2,46871219	1,14354018	1,27483652	1,8305466	1,63600425	1
7	0,98221556	1,53799839	1,73875299	2,77085502	1,8090151	1,28267415	1,28465219	1,4600972	0
8	2,18942543	1,94196854	2,78170334	3,54103499	1,64354076	1,48817338	2,67496409	2,61560743	0
9	3,54931685	3,45958771	3,2486703	4,36630642	1,68634468	1,61180779	1,72136181	2,43467565	1
10	2,7826023	1,74404483	1,44436445	2,64068344	0,84904281	1,06024755	0,9772857	1,6507413	1
11	2,17519292	1,75120039	2,82733023	2,23648337	1,78530792	1,32600867	2,29511211	1,65292078	0
12	1,644514	1,170473	2,229521	2,294323	2,593279	1,011488	1,899258	1,280298	0
13	1,3676728	1,78151593	1,59474499	3,41163659	1,77688738	1,0816034	1,6046543	1,75094079	0
14	1,16856435	0,82438059	1,37685569	1,89526496	0,81602805	0,61157073	0,85136351	0,82734918	2
15	1,10607607	1,75766945	1,85412034	2,24925461	1,51577194	1,04240682	2,13437407	1,4389085	3
16	1,88289735	2,18349202	2,50702203	2,20622896	1,54614571	0,57793901	2,22458457	1,85937205	1
17	1,48801304	1,84093917	1,88534744	5,06723912	2,14161685	1,39299719	1,80656442	2,04602745	0
18	0,86475842	1,08520523	1,16957301	2,47873282	1,3310987	0,8698512	1,26094985	1,30112781	1
19	1,88416235	1,97070574	2,24832877	4,06482005	2,82794266	1,87087139	2,09715831	2,57164233	0
20	1,4445008	1,24905857	1,55505949	2,35917311	1,16902858	0,91864644	1,34813195	1,48747805	0
21	1,18639776	1,20943745	1,72875009	2,57818866	1,1236604	0,87047588	0,99947799	1,03917666	1
22	1,25688703	1,15383614	1,07612784	1,73737752	0,93020045	0,82246499	1,24799209	1,46979649	1
23	1,40429242	1,25195012	2,52613948	1,88280029	2,74459883	1,25270073	1,98247266	1,23970891	1+2
24	1,663232	1,753679	2,004716	4,85057	2,023793	1,577285	1,776929	1,842059	0
25	1,767255	3,152258	1,506168	4,287804	1,485321	1,661794	1,546511	2,20309	0
26	1,287718	1,23489334	1,97392725	1,91937635	1,10465331	0,73496577	1,40388226	1,01743102	2
27	1,31973089	1,24954251	2,05257478	2,79112811	1,29189821	1,04969635	1,45361605	1,38295335	0
28	1,61408203	1,56296296	1,55950865	4,00347601	1,43013212	1,29501918	1,37536465	1,75008681	1
29	0,714545	1,38301	1,156303	2,321721	0,856403	0,914265	1,10681	1,475072	0
30	1,89846489	2,31185582	2,29808631	6,23995985	2,62791371	1,82782226	2,18875411	2,33423841	0
31	1,32460432	1,4879307	1,30281053	2,69330317	1,01952809	0,93195712	1,19177898	1,29227502	0
32	0,72632289	0,81848691	0,97984413	1,62133661	1,0478258	0,62363931	0,63943345	0,84925004	1
33	1,785231	2,685112	2,914858	5,515835	2,152856	1,757714	1,993525	1,872959	0
34	1,28677518	0,95231477	1,13842677	1,80541587	1,09550238	0,94697559	1,1193422	1,34848305	1+2

Results were expressed in Relative Quantification (RQ); results were the median of three individual measurements. Ongoing Therapy: 0 = No treatment; 1 = Eltrombopag; 2 = MMF; 3 = Sirolimus

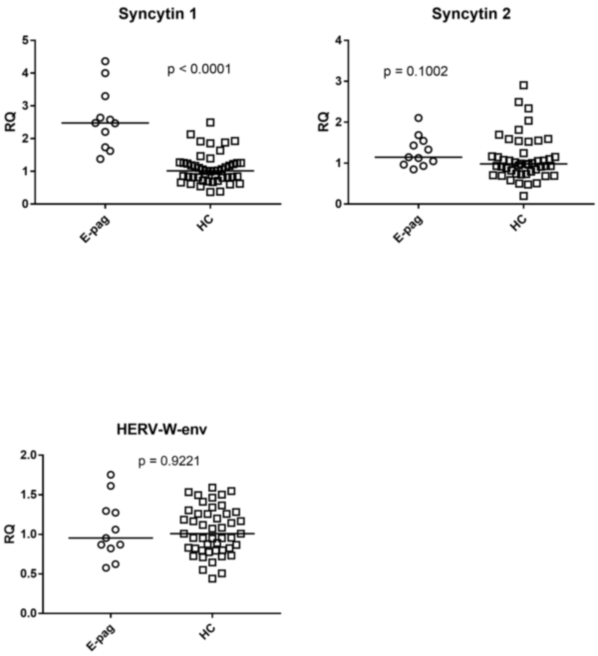

Supplementary Figure S1

Transcription levels of env genes of Syncytin 1, Syncytin 2 and HERV-W in whole blood from 11 patients on Eltrombopag treatment alone (E-pag) and 47 healthy controls (HC).

RQ: Relative Quantification. Circles and squares show the median of three individual measurements, horizontal lines the median values. Statistical analysis: Mann-Whitney test was used to compare the transcriptional levels of each gene.

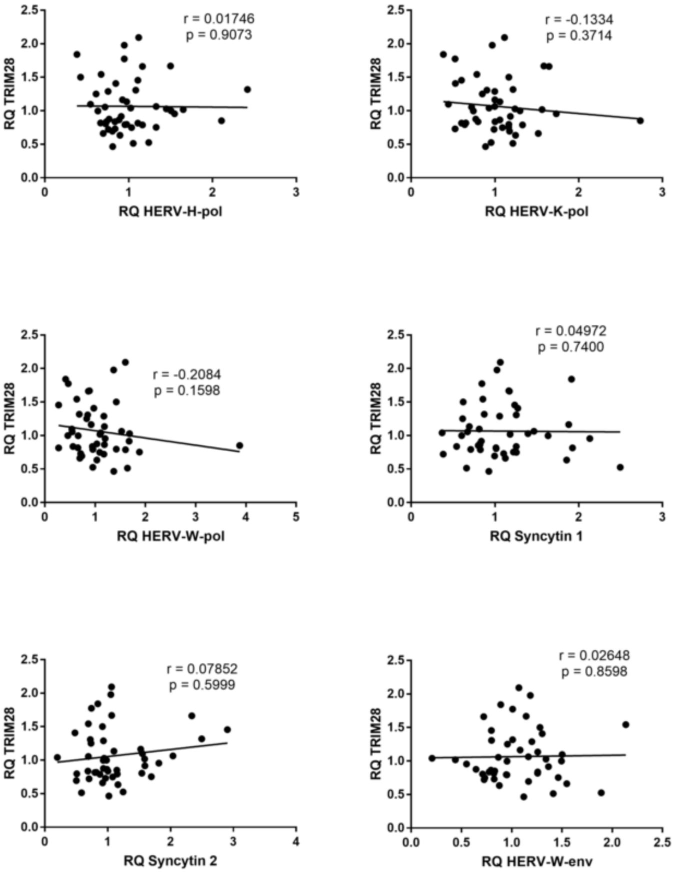

Supplementary Figure S2

Correlations between transcription levels of TRIM28 and HERV sequences in whole blood from healthy controls (HC).

RQ: Relative Quantification. Circles show the mean of three individual measurements. Line: Linear regression line. Statistical analysis: Spearman correlation test.

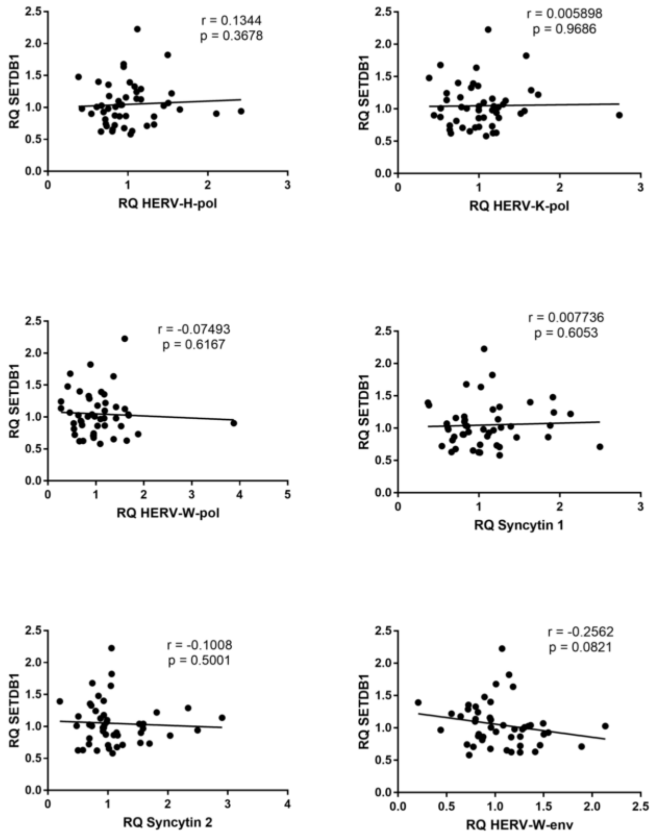

Supplementary Figure S3

Correlations between transcription levels of SETDB1 and HERV sequences in whole blood from healthy controls (HC).

RQ: Relative Quantification. Circles show the mean of three individual measurements. Line: Linear regression line. Statistical analysis: Spearman correlation test.

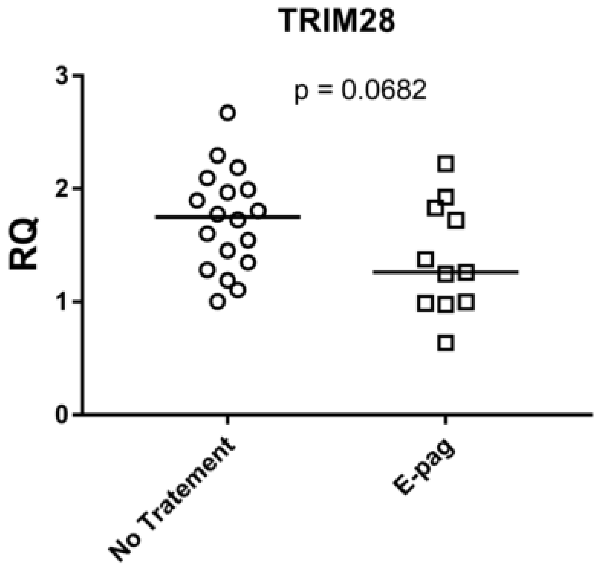

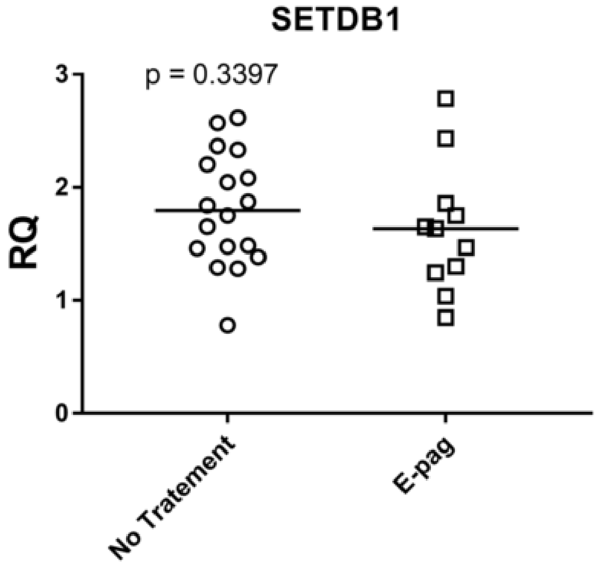

Supplementary Figure S4

Transcription levels of TRIM28 and SETDB1 in whole blood from 11 patients on Eltrombopag treatment alone (E-pag) and 18 patients with no treatment.

RQ: Relative Quantification. Circles and squares show the median of three individual measurements, horizontal lines the median values. Statistical analysis: Mann-Whitney test was used to compare the transcriptional levels of each gene.
